# A virtual dance sport class for cancer patients: the trainer perspective

**DOI:** 10.1007/s00432-022-03913-6

**Published:** 2022-03-07

**Authors:** Christian Keinki, Ivonne Rudolph, Tobias Wozniak, Ronny Pietsch, Mascha Margolina, Isabel Garcia, Katharina Mayr-Welschlau, Thorsten Schmidt, Jutta Hübner

**Affiliations:** 1 Medizinische Klinik II, Hämatologie und Internistische Onkologie, Universitätsklinikum Jena, Am Klinikum 1, 07747 Jena, Germany; 2 Waldburg- Zeil Kliniken, Rehabilitationsklinik Bad Salzelmen, Badepark 5, 39218 Schönebeck, Germany; 3 Working Group Prevention and Integrative Oncology, German Cancer Society, 14057 Berlin, Germany; 4 Cancer Center North, University Hospital Schleswig-Holstein; Campus Kiel, Arnold-Heller-Straße 3, 24105 Kiel, Germany

**Keywords:** Neoplasm, Physical activity, Dancing, Quality of life, Virtual training

## Abstract

**Purpose:**

Due to the corona, pandemic classes with physical activity for cancer patients were postponed. For an ongoing program with ballroom dancing classes for patients and their partners, the training was switched to a digital format.

**Methods:**

We evaluated the training by structured written interviews of the trainers including an open report part concerning the development and realization of the project, the teaching and training concept and their experiences as trainers.

**Results:**

5 trainers reported data from 6 different classes including 65 participants. All in all, digital dance training is feasible and a substantial part of the participants of former face-to-face training took part. Yet, digital training imposes some restrictions to the movements taught and the interactions with and between the participants. Trainers have to invest time in a new conception of the training and additional time in guiding participants with lower experience in technical issues.

**Conclusion:**

Participants in virtual training rooms need more support and social interactions in digital training are less and different from ballroom lessons and trainers which puts more strain on trainers to motivate cancer patients.

## Introduction

Physical activity is of upmost importance during and after cancer treatment. Yet, one decisive key is the motivation of the participants. Physical active improves coping, reduces side effects of treatments and improves quality of life (Duijts et al. 2011; Bourke et al. 2016; Oberoi et al. 2018; Browall et al. 2018). Moreover, physical activity also has a strong impact on prognosis (Friedenreich et al. 2020). Accordingly, guidelines give a strong recommendation on regular training for all patients and survivors (Campbell et al. 2019; Leitlinienprogramm Onkologie (Deutsche Krebsgesellschaft, Deutsche Krebshilfe, AWMF 2020).

In 2016, we started with a ballroom dancing project for cancer patients and evaluated the program in several studies. Dancing helps to integrate the partner and is perceived more as a social activity than a strenuous effort by the participants. Experienced trainers show the participants how to tailor the intensity of the movements so that patients may take part even on days with less physical forces or more fatigue (Rudolph et al. 2018, 2021; Schmidt et al. 2018).

Dance movement therapy has physical, mental and social benefits and has been evaluated mostly as individual dancing (Mannheim et al. 2013; Lemercier et al. 2014; Szalai et al. 2015; Ho et al. 2016; Butler et al. 2016). As elaborate assessments (Kattenstroth et al. 2013; Pisu et al. 2017) and a high number of hours of training per week have been shown to reduce adherence, we offer a low level, highly motivating open course once per week continuously over the whole year (Rudolph et al. 2018, 2021; Schmidt et al. 2018). A broad range of dancing for couples (Standard and Latin-American) included different music styles. Thus, most participants feel familiar with the music and the dancing movements. Evaluations have shown the feasibility of this training and provide data on improving well-being and fatigue (Thieser et al. 2021).

The project was expanded to three more cities till in 2020, the corona pandemic forced us to close the groups. All trainers are experienced trainers on ballroom dancing for adults, some have additional courses as trainers for disabled persons. Concerning cancer patients, they got a theoretical and practical introduction including one workshop with patient training and one theoretical workshop on cancer, communication with cancer patients, cancer treatment and side effects and possible adverse events during training classes.

During the Corona pandemic, physical activity in the general population went down. This was also true for cancer patients (Schmidt et al. 2021). While there are first data on virtual training for cancer patients with different types of home exercises and monitoring (Dong et al. 2019), no data exist so far on a virtual dance training.

As Germany went in a further lockdown in January 2021, we decided to restart the classes virtually and asked all trainers to develop a concept and invite the former participants. Moreover, we planned a new group with one trainer. In parallel, we prepared the evaluation of the digital courses and the concepts, to learn more about feasibility, chances and barriers of a digital ballroom dance course for cancer patients. In this article, we describe the data from the assessment of the concept and experiences of the trainers, while the data from the patient evaluation will be presented in a second manuscript that is currently being prepared. 

## Methods

### Participants

All five trainers of the dance sport project were addressed whether they would start a virtual class. Trainers consenting were asked to develop a training concept and to offer one training per week to all former patients and partners. One trainer also consented to offer a course for beginners.

All trainers have worked with cancer patients for at least 1 year before. While one trainer (TW) started the project in cooperation with a physician (JH) and a rehabilitation scientist (IR) 5 years before, the others were introduced theoretically and practically in at least one workshop and attended a workshop on training and communication with cancer patients by the working group PRIO.

### Training

The courses were open to patients during and after cancer treatment or in case of relapse and consequent treatment. Training was offered once a week using a digital platform with login by simple mail without password. The system was either jitsi hosted on a server regarding all aspects of data safety or zoom. Patients were asked to activate their camera for better corrections by the trainer but were free to reject this.

The diagnosis or handicaps of the attending patients were not asked by the trainer due to data protection reasons. To avoid pain or overexertion, all participants are told to move in their respective physical possibilities. Since no adverse effects occurred during the piloting of the project, no accompanying evaluation of the state of health was carried out during the digital courses (Schmidt et al. 2018). In the event of uncertainties, the participants were asked to consult the attending physicians.

### Evaluation

The evaluation included a period of 12 weeks from the first lesson ongoing.

We evaluated the training by structured written interviews of the trainers including an open report part concerning the development and realization of the project, the teaching and training concept and their experiences as trainers. The interview consisted of six sections:

1. Former experience with digital dance classes
2. Concept of the training (dancing style, dancing as single or pair, transformation of the normal dance room class or new concept)
3. Time and effort needed for the new concept
4. Implementation of the course
5. Technical assistance needed by the participants
6. Comparison to a training in the dance room

Patients were asked to fill in a structured questionnaire, which was different from that of the trainer. Data of the patients are described elsewhere (Hübner et al.).

### Statistics

We used IBM SPSS Statistics 27 for the data collection and statistical analysis. For associations, we used the Chi-Square test with *p* < 0.05 being considered as significant.

### Ethics vote

The study was approved by the ethics committee of the university hospital at Jena.

## Results

All five trainers (three female, two male) consented to participate, answered the questions, and returned a detailed concept. All trainers were experienced trainers for ballroom dancing with several years of training experience with adults and at least 2 years of experience in training with cancer patients (maximum 6 years).

### Results of the structured questionnaire

Considering own experience in digital formats, three answered to have taught online themselves and two had taken part in online-courses. For four trainers, the decision to offer a virtual format came easy, while one reported having considered pros and cons more in detail. All five included ballroom dances in their concept (Standard and Latin) and four included Line Dance. Four trainers reported simplifying the movements in comparison to the normal classes and only one used the same steps and movements. Three included new steps/movements while two did not.

Two trainers enriched their classes with additional online material (videos showing the movements), two did not.

Time needed for preparation of the digital concept was heterogeneous from 1 to 2 days to 3 to 4 weeks, but three documented that it took only 2–3 h all in all, while two reported more than 10 h. For preparing the next lesson, they needed between 10 and 120 min. Three of the five trainers marked that preparation was more difficult than in normal classes and that planning and foreseeing the lesson was as easy as in normal classes.

All trainers used a laptop or tablet with WLAN or LAN, which to their knowledge was also the most often used access by patients. Considering the platform, only one trainer was fully satisfied, four reported that it was stable but sometimes of low quality. This was true for jitsi as well as zoom. All in all, four of the five trainers reported that the technical preparations were easy or rather easy.

All trainers had invited the participants by mail, four also by telephone call and several mails or calls were necessary to motivate some patients. In comparison to the former number of participants, they were able to recruit 60 of 104 former participants of the classes (57.7%). Three trainers marked that convincing the patients and partners to join the virtual training was easy, while two thought that it was a challenge.

With respect to the technical procedures, 12 patients (11.5%) needed support before the first class by the trainer and another 12 patients (11.5%) reported help form members of the family. Thirteen patients (12.5%) needed technical support during the first class by the trainer and 6 patients (5.8%) needed support several times.

The duration of the training varied from 60 (four trainers) to 75 min (one trainer) with an additional few minutes of small talk at the beginning and at the end. While two trainers did not include a pause, three planned a pause from 1 to 10 min.

As a summary, four trainers consented that the virtual training was fun to them. Yet, four also said, that it was less or much less fun than the real-life classes. While selection of the music was as easy as in normal classes, showing and controlling the movements was more difficult (four trainers each) and all missed the direct contact to the patients. Two trainers believed that they were less able to judge the well-being of the participants, three did rather not consent. All in all, even after the pandemic, one trainer strongly voted for continuing the digital training, two would consider it and two voted against it.

### Concepts of the trainers

The concepts between the trainers varied to some extent. All included elements from Standard and Latin ballroom dancing. Most also included Line Dance elements. As many patients took part without a partner, movements for couples were adapted accordingly. Moreover, the steps were adapted to fit in small room of about 1 square meter.

While all trainers developed their concepts independently, there is a common scheme. The lesson starts with a warm-up, using movements and steps which are already familiar. This phase also was a training of coordination and balance. Some trainers also included stretching. In the second phase, choreographies were repeated, adapted and steps added. A third phase concentrated on new movements and steps or on working on more exactness of the movements. In a last cool down, relaxation, mindfulness and coordination were the dominant elements.

All trainers reported explicitly to address condition, motor skills, most also mentioned elasticity, balance or strength. For all trainers, participants having fun was a central goal. A detailed plan for one lesson is presented in Table 1.

**Table 1:** Plan for one typical digital dancing lesson

Time in minutes	Type of dancing	Content
10	Warm up—movements to music	Warming up of muscles; coordination, agility and balance
30	Linedance	Repetition of a choreography, work on difficult steps New choreography
30	Ballroom dancing Standard (Valse) Latin (Cha Cha Cha, Rumba) Other dances (Discofox, Boogie, Mambo)	Repetition of learnt steps and figures as single or pair New steps or figures New dance (f. ex. Jive)
10	Cool down	Attention and mindfulness, breathing, stretching

## Discussion

The implementation of a virtual dance movement training for cancer patients with a focus on Standard and Latin dances is feasible and finds rather high acceptance by participants with experience in real-life classes. Yet, for trainers, some special preparations are important and the concept has to be adapted for several reasons. The time needed for these preparations differs by experience with digital formats. Moreover, trainers who included line dance elements seem to need less time as those who tried to adapt in detail ballroom dancing movements for most of the time.

One problem is the web-based technical access which still is a barrier for many older people but also for younger ones especially if platforms are used which they are not familiar with. In contrast, nearly all trainers of our study had own experiences with online training lessons either as participant or as trainer. They were able to provide support before the first online class and even during lessons. A few patients needed this support not only once.

The second problem is the missing place in private lodgings. As the trainers stated at least 1.5 square meters is needed for one person. Latin dancing also in a couple may be performed in a small place for simple figures but standard dancing as pair needs much more space. While mostly, the trainers were able to use a training room of their dancing school or association, using a private room not only reduces the area but also may provide insight into privacy. This might also be a problem for the participants as not only the trainer but also other participants may look into rooms. For trainers, it is not possible to turn down the camera. Some patients used this option, but this reduces the possibility of the trainer to give advice and correct. As the trainers have to watch several participants, this is a challenge on a tablet or laptop, which could be improved using large screens.

In fact, while music does not seem to be a problem in comparison to the real-life classes, the visual contact to the group is hindered and not all trainers are sure whether they are able to recognize the well-being of a participant as easily as during real-life classes.

Less area for dancing is one reason why the steps have to be adapted especially for Standard Dancing which takes much room. More difficulties to be watched by the participants and to watch participants for correction are the second one. Moreover, direct help by leading a trainee is not possible in a virtual classroom.

Another reason for adaptations was that a majority of patients had no partner taking part in the lessons. In real-life classes, two singles may dance together which during the lockdown only was possible with other family members. Accordingly, all movements have to be dance-able separately. This is the reason why Line Dance as a dance with every person making his/her own movements in the group was integrated into the lessons more than during real-life classes.

The concepts developed independently by five experienced trainers for dancing with cancer patients are quite similar with one lessen lasting about 60 min with an additional few minutes for talking. Structured longer pauses are not necessary which is quite in contrast to the real-life classes which most often last about 90 min with one pause of 10 min in the middle. Warming up and cooling down are the start- and stop-phase of all concepts, with some differences whether the focus is on stretching, coordination or first steps. New movements or choreographies are in the middle of the lesson to be most flexible for the time it takes to explain and train and to have the full attentiveness of the patients.

Dancing not only addresses several characteristics of movements as endurance, strength, coordination, elasticity and balance and thus offers a broad range of training effects. Besides, it normally is a social activity. The lack of direct contact was one reason of doubts on the feasibility also for the patients. Motivating them to join the online courses was time consuming and needed several attempts in a majority. Nevertheless, all trainers were able to recruit more than half of the former participants, in spite nearly 1 year having past since the last class in presence.

Despite all obstacles, four of five trainers had fun during the lessons. Yet, it is less fun than in the real-life classes, which most prominently is due to the missing direct contact. Accordingly, their opinion on offering virtual classes after the Corona pandemic is split. The strictly positive vote for an ongoing virtual offer came from the trainer who started a course with beginners during the study. It might be that the fact that the participants did not know the former atmosphere and were recruited from a telemedical consultation facility increased acceptance and active participation which makes it much easier for the trainers. The advantage of this offer is, that it can be brought to any town or village thus including patients from structurally weaker and even rural areas.

The parallel assessment of the effects on well-being of the patients shows a strong effect over the weeks (Hübner et al.)—to find out whether this is similar to real-life classes, the same study is repeated by us now after the restart of classes in presence.

While feasibility has been shown, we must keep in mind that online training is multitasking for the trainers, more time consuming and more exhausting. Support for example for the technical problems might reduce some stress.
